# Bragg Coherent Diffractive Imaging of Zinc Oxide Acoustic Phonons at Picosecond Timescales

**DOI:** 10.1038/s41598-017-09999-0

**Published:** 2017-08-29

**Authors:** A. Ulvestad, M. J. Cherukara, R. Harder, W. Cha, I. K. Robinson, S. Soog, S. Nelson, D. Zhu, G. B. Stephenson, O. Heinonen, A. Jokisaari

**Affiliations:** 1 Materials Science Division, Argonne National Laboratory, Argonne, Illinois 60439 USA; 2 Advanced Photon Source, Argonne National Laboratory, Argonne, Illinois 60439 USA; 3 Condensed Matter Physics and Materials Science Department, Brookhaven National Laboratory, Upton, New York, 11973 USA; 4 London Center for Nanotechnology, University College London, London, WC1E 6BT United Kingdom; 5 LCLS, SLAC National Accelerator Laboratory, Menlo Park, California, USA; 6 Northwestern University, Evanston, Illinois 60208 USA

## Abstract

Mesoscale thermal transport is of fundamental interest and practical importance in materials such as thermoelectrics. Coherent lattice vibrations (acoustic phonons) govern thermal transport in crystalline solids and are affected by the shape, size, and defect density in nanoscale materials. The advent of hard x-ray free electron lasers (XFELs) capable of producing ultrafast x-ray pulses has significantly impacted the understanding of acoustic phonons by enabling their direct study with x-rays. However, previous studies have reported ensemble-averaged results that cannot distinguish the impact of mesoscale heterogeneity on the phonon dynamics. Here we use Bragg coherent diffractive imaging (BCDI) to resolve the 4D evolution of the acoustic phonons in a single zinc oxide rod with a spatial resolution of 50 nm and a temporal resolution of 25 picoseconds. We observe homogeneous (lattice breathing/rotation) and inhomogeneous (shear) acoustic phonon modes, which are compared to finite element simulations. We investigate the possibility of changing phonon dynamics by altering the crystal through acid etching. We find that the acid heterogeneously dissolves the crystal volume, which will significantly impact the phonon dynamics. In general, our results represent the first step towards understanding the effect of structural properties at the individual crystal level on phonon dynamics.

## Introduction

In crystalline solids, atomic positions deviate from their equilibrium positions under external stimuli such as strain and temperature. The transient, collective excitations of atomic motion are known as phonons, and acoustic phonons are the excitations in which all atoms in a given unit cell move in the same direction. Acoustic phonons play an important role in many physical phenomena including phase transitions^1^, electron energy relaxation in semiconductors^2^, and transient melting^3^. The advent of x-ray free electron lasers (XFELs) has opened considerable new avenues for investigating both optical and acoustic phonon properties utilizing pump-probe techniques^4, 5^. Typically, ultrafast optical pulses are used to induce phonons through electron phonon coupling. In semiconductors, the light produces electrons and holes that emit phonons and relax to band edges in 1 ps^6^. Recombination of the electrons and holes occurs on a longer time scale (1 ns to 1 μs). The spatial distribution of the phonon modes and their properties can be changed by tuning parameters such as crystal size and shape, surface roughness, and defect density^7–9^. Structures on the order of nanometers to micrometers in particular offer considerable freedom in designing properties for various applications.

While individual crystals offer freedom for tuning material properties^10–12^, understanding the connection between a particular structure and the resulting function remains challenging due to the short time and length scales of interest. Consequently, many studies rely on particle ensembles that average over the mesoscale heterogeneity, which makes it difficult to elucidate the true structure-function relationship^13^. For example, ensemble-averaged studies can conclude that relaxation times are shorter than they are in individual particles^14^. Imaging of single particles has been achieved using optical techniques^13^, but these cannot probe atomic scale motion due to their long wavelength. Bragg coherent diffractive imaging (BCDI) with hard x-rays^15, 16^ is uniquely suited to probing atomic motions, thereby resolving acoustic phonons, at the individual crystal level with nanometer resolution. While other techniques couple indirectly to phonon motion, for example through changes in optical properties^6^ or through plasmonic properties^12^, BCDI directly images the atomic displacements and thus the phonons. Ultrafast BCDI at an XFEL was recently used to resolve acoustic phonon modes in a gold crystal^3, 17^. In this article, we use ultrafast BCDI to visualize the 3D acoustic phonon dynamics in single zinc oxide rods at ultrafast timescales using an XFEL.

In BCDI, the 3D intensity distributions around a Bragg peak are collected by slightly rotating (by ~0.2°) the crystal with respect to the incident x-rays. The 3D intensity distribution is inverted into a real space image via iterative phase retrieval algorithms^18–21^. The real space image is complex: the amplitude is proportional to the diffracting or Bragg electron density^22^ and the phase is proportional to a projection of the atomic displacement field onto the measured scattering vector^15, 23, 24^. The Bragg electron density can be used to identify crystal regions of a different phase^25, 26^ while the atomic displacement field can be used to identify dislocations and reveal the strain distribution^26–31^. When BCDI is used stroboscopically in a pump-probe experiment, the temporal resolution for repeatable dynamics is limited by the pulse widths of the laser pump and the x-ray probe. For further details please see the Methods.

## Results

**Figure 1:**
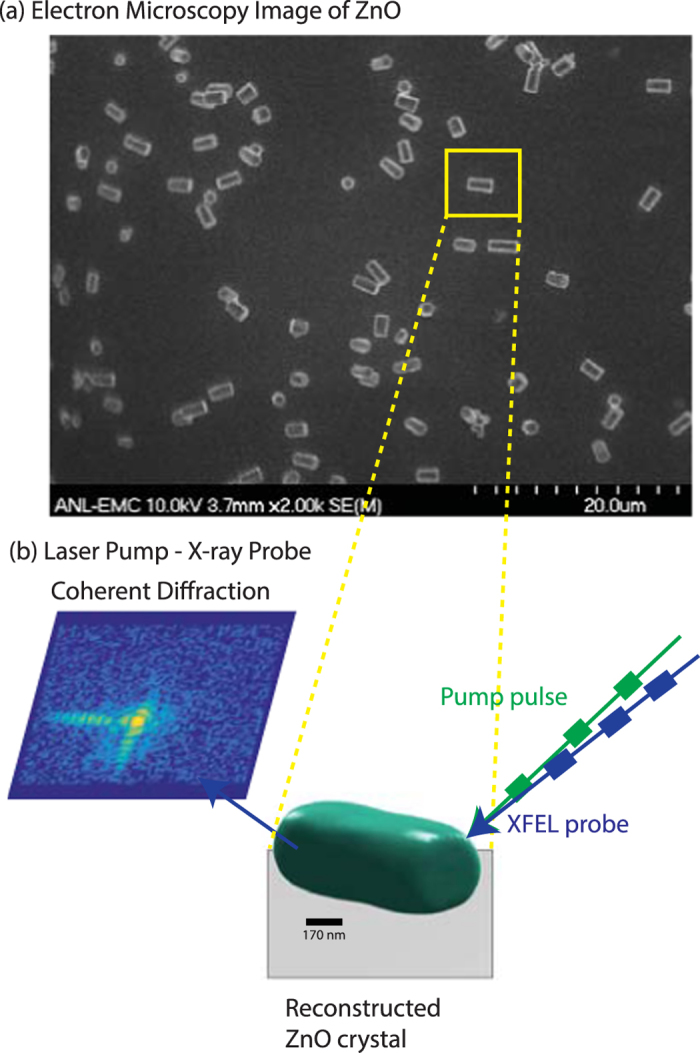
Schematic of the LCLS XPP laser pump - x-ray probe experiment^4, 32^. (**a**) Electron microscopy image of the as-synthesized ZnO rods. The crystals show the hexagonal shape expected for the wurtzite phase and range in size from 500 nm to 1.5 micron in length. **(b)** 2D cross-section of the 3D experimental diffraction data from an individual ZnO crystal that is isolated on the x-ray sensitive CS-PAD area detector. The diffraction data at each delay time is recorded stroboscopically for 1000 shots at 60 different angles, which then forms the 3D diffraction data. Iterative phase retrieval is used to reconstruct the image of the ZnO crystal (shown as a green isosurface). The crystal is approximately 640 nm long by 580 nm in the other two dimensions. 3D coherent diffraction data sets are recorded for different delay times.

ZnO rods of different shapes, sizes, and aspect ratios were prepared via a chemical vapor deposition process (see Methods, Fig. 1a). The different orientations of the crystals on the substrate allow Bragg peaks from individual crystals to be isolated on an x-ray sensitive area detector (Fig. 1b). The excitation of acoustic phonons in the ZnO crystal by the laser pulse can be seen directly in the coherent diffraction data.

**Figure 2:**
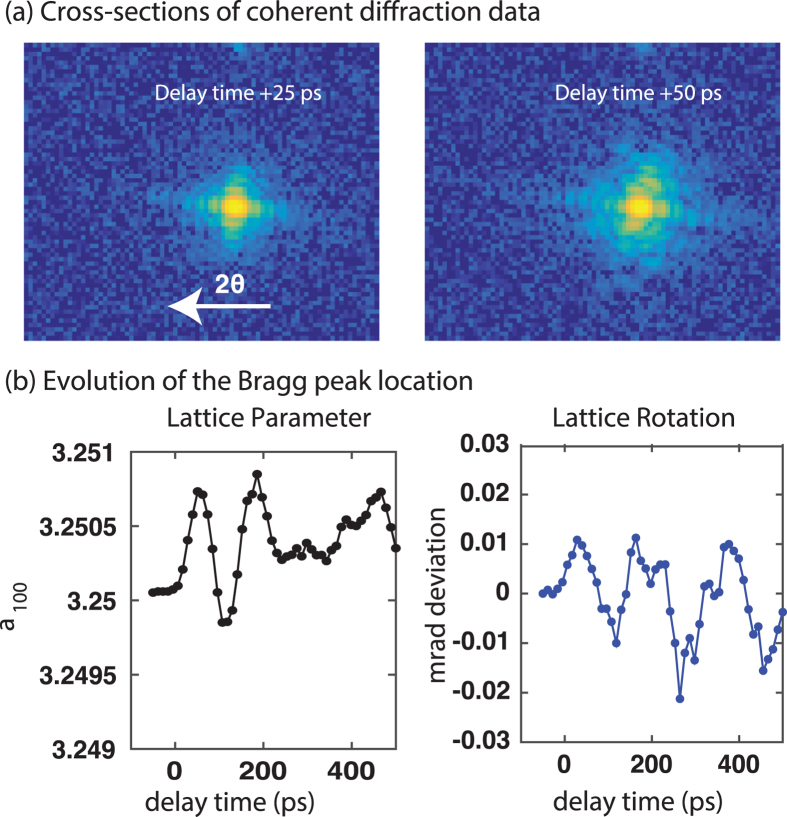
Homogeneous acoustic phonon mode excitation in a single ZnO crystal. (**a**) The (100) Bragg peak from a single ZnO crystal at two different delay times (+25 and +50 ps). Positive delay times correspond to the laser pump followed by the x-ray probe. The pump induces distortion to both the Bragg peak location and its intensity distribution at +50 ps. (**b**) Analysis of the horizontal (lattice parameter evolution) and vertical (lattice rotation) Bragg peak location as a function of delay time. The uncertainty is represented by the symbol size. The dominant frequencies in the two oscillations are 7 GHz and 5.5 GHz.

Coherent diffraction data were collected from a single ZnO crystal for a number of delay times from −50 ps to +500 ps (Fig. 2a, Supplementary Movie 1). For the chosen experimental geometry, the horizontal direction on the detector is in the scattering vector direction while the Debye-Scherrer ring is in the vertical direction. The laser pump induces significant distortion to the Bragg peak at positive delay times. Note that we observe no change in the Bragg peak position at negative delay times, indicating that any dynamics induced by the laser fully relax before the arrival of the next pair of pulses. This is in contrast to a previous study^33^ that used a synchrotron source and consequently a much higher repetition rate (6.5 MHz in that study vs. the 120 Hz of this study). The generation of acoustic phonons changes the Bragg peak location in both the horizontal and vertical directions. The Bragg peak center was extracted using Gaussian fits to the sum of the intensity in each direction. The (100) lattice parameter changes by a maximum of approximately 1 × 10^−3^ Å, corresponding to a temperature change of 35 °C. Average lattice expansion and contraction, as observed in Fig. 2b, is a breathing mode: the crystal expands and contracts everywhere uniformly. The dominant frequency in this breathing mode is approximately 7 GHz, which matches with the average speed of sound in wurtzite ZnO^34^. The Bragg peak location also changes in the vertical direction, which corresponds to a rotational homogeneous deformation mode of the (100) lattice planes. The dominant frequency in this mode is 5.5 GHz. Note that the properties of these modes are a direct consequence of the finite size of the crystal.

By analyzing the coherent diffraction data from a single ZnO crystal during a pump-probe experiment, we have observed two homogeneous phonon modes: a breathing mode with a 7 GHz frequency and a rotational mode with a 5.5 GHz frequency. These results are consistent with acoustic phonon mode excitation in the ZnO crystal through laser excitation of electrons followed by electron-phonon coupling. While the homogeneous modes reveal the average behavior throughout the crystal, a unique strength of BCDI is the ability to reveal the 3D inhomogeneous phonon dynamics. To obtain these images, 60 2D slices of the 3D Bragg peak are taken at delay times from −25 to +300 ps in 25 ps steps and used to reconstruct real space images through iterative phase retrieval. The pixel size of the reconstructed images is 17 nm and the resolution is estimated at 3 pixels (51 nm) from the sharpness of the reconstructed particle boundary.

**Figure 3:**
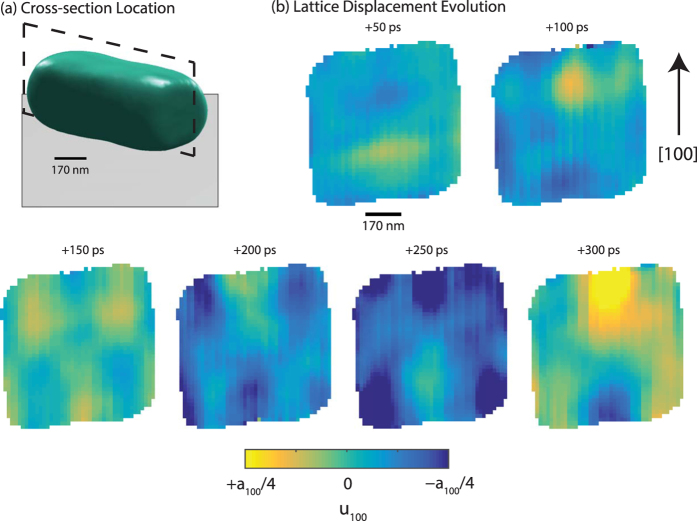
Local displacement field evolution in an individual ZnO crystal during laser excitation. The displacement field component, *u*_100_(x, y, z), throughout the particle is shown at a cross-section in the particle shown by the black dashed plane in (**a**). The ZnO crystal is approximately 640 nm long by 580 nm in the other dimensions. The grey plane represents the substrate. The [100] direction is vertical in the images in (**b**) and shown by the black arrow. The following states are shown: +50, +100, +150, +200, +250, and +300 ps. The scalebar and colorbar apply to all images.

Figure 3a shows the spatial location of the cross-section in the particle at which the *u*_100_ displacement field distribution is shown in Fig. 3b. The colormap represents the displacement in the [100] direction of the atoms from their equilibrium positions, which we define by the average lattice constant. At +50 ps, we observe primarily two regions of large displacement: one positive and one negative. At +100 ps, the sign of these two regions has reversed (positive has become negative and vice-versa). At +150 ps, a higher order displacement field distribution is observed with alternating regions of positive – negative – positive at the top and bottom of the cross-section. These regions alternate in sign 50 ps later (at +200 ps). The signs reverse again at +250 and +300 ps. These images show the spatial distribution of shear acoustic phonon modes inside the ZnO crystal. We observe regions of alternating positive and negative displacement, and an increasing in the order of the phonon mode for longer delay times.

**Figure 4:**
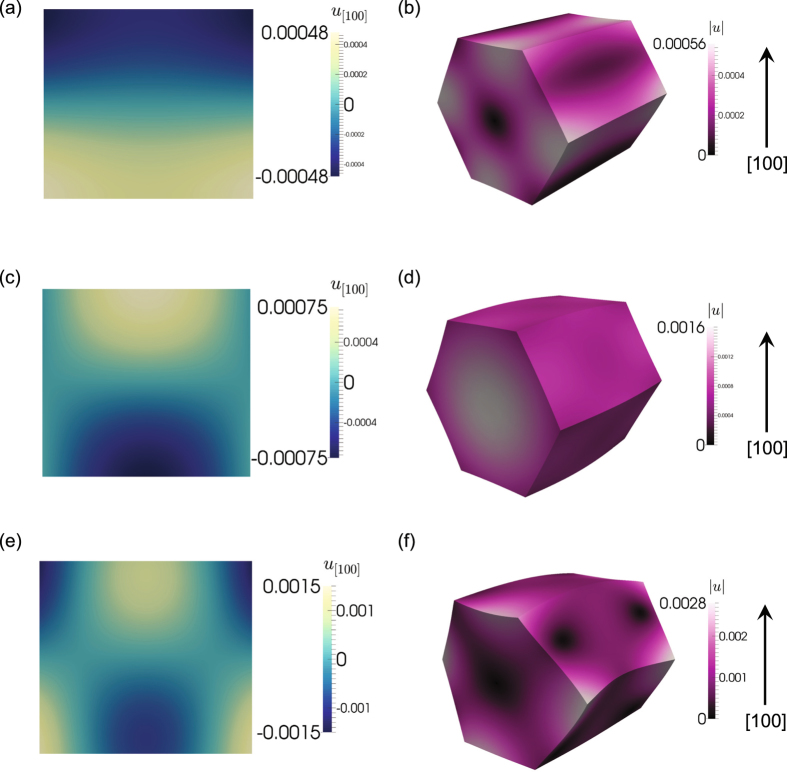
The calculated displacements for modes with 3.7 (**a**,**b**), 3.9 **(c**,**d**), and 4.0 (**e**,**f**) GHz frequencies. (**a**,**c**,**e**) The calculated displacement field component, *u*_100_, in the same cross-section as for that in the experimental measurements. (**b**,**d**,**e**) The ZnO nanoparticle is warped by the total modal displacements $(|{\boldsymbol{u}}|)$ to visualize the phonon mode type (warp factor of 2 × 10^5^ to make distortions evident).

To interpret the inhomogeneous phonon dynamics, we compare the observed displacement fields with those calculated via finite element simulations of acoustic phonon modes (see Methods for further details). The frequencies for the 16 lowest eigenmodes are 2.3, 3.1, 3.2, 3.3, 3.7, 3.9, 4.0, 4.4, 4.6, and 4.7 GHz (several different modal patterns exhibited the same frequencies). To understand what modes are present, the calculated and measured *u*_100_ displacement field components in the same particle cross-sections are compared. Figure 4 shows the calculated displacement for both the *u*_100_ component and $|{\boldsymbol{u}}|$ for the 3.7 (Fig. 4a,b), 3.9 (Fig. 4c,d), and 4.0 (Fig. 4e,f) GHz frequencies. The *u*_100_ displacement component for the modes with 3.7 and 3.9 GHz frequencies is similar to that seen in the +50 and +100 ps snapshots in Fig. 3b, while the *u*_100_ displacement component for the mode with the 4.0 GHz frequency is similar to that seen in the later snapshots at +150, +200, +250, and +300 ps. The displacement fields for the other calculated phonon modes are not similar. Visualizing the total displacement of the particle $(|{\boldsymbol{u}}|)$ shows that these are transverse modes, in which the particle expands and contracts at the 3.7 and 3.9 GHz frequencies, while it bends at the 4.0 GHz frequency.

The ability to image the shear acoustic phonon modes in 3D is a unique feature of BCDI and, in principle, can be used to track the evolution of phonon modes with crystal size and shape. To this end, we performed synchrotron BCDI measurements to elucidate the effect of acid etching on the ZnO crystal shape and size (see Methods for further details).

**Figure 5:**
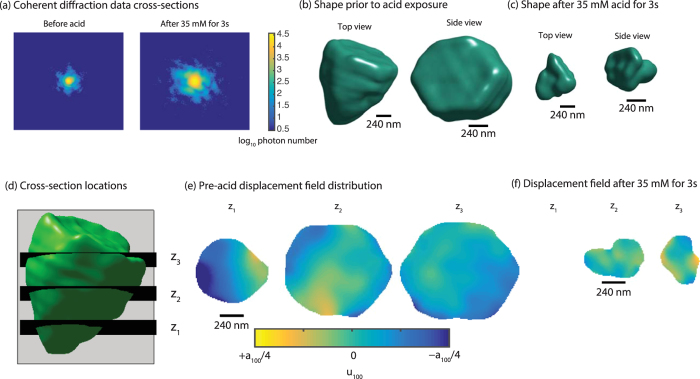
Demonstration of tracking shape modification in an individual ZnO crystal during dilute nitric acid exposure. (**a**) The central cross-section of the 3D coherent diffraction data before and after acid exposure. The increase in fringe size corresponds to a decrease in particle size. (**b**) The reconstructed shape for two different views before acid exposure. (**c**) The reconstructed shape for two different views after acid exposure. Acid exposure significantly reduces the crystal volume. (**d**) The cross-section locations for the cross-sections shown in (**e**) and (**f**). (**e**) The displacement field distribution at three cross-sections before the acid exposure. (**f**) The displacement field distribution at the same cross-sections after acid exposure. The displacement field distribution has significantly changed.

Figure 5 shows the changes observed in a ZnO crystal due to acid etching. The as-synthesized state was characterized by collecting 3D intensity distributions. Then, 35 mM nitric acid was added dropwise to the sample (see Methods for further details). Figure 5a shows this caused significant changes to the coherent diffraction data. The fringe size, which scales inversely with the crystal size, has increased, indicating the particle has decreased in size. Figure 5b and c shows the reconstructed crystal images that confirm the size reduction. With the 3D information, we show the displacement field distribution at 3 different cross-sections (Fig. 5d–f). Their spatial location relative to the isosurface is shown in Fig. 5d. The initial displacement field has regions of magnitude a_100_/4. The acid exposure causes significant changes to the displacement field distribution (Fig. 5e–f), but does not induce any dislocations^27, 28, 35^ or a change in surface roughness that is visible with the 50 nm resolution. Based on these results, we conclude that acid exposure can be used to investigate the influence of size and shape on the excited phonon mode distributions, but cannot be used to induce dislocations or change the surface roughness. This is important for the design of future experiments. The degree of etching can be controlled through a combination of the acid strength and exposure time. While shape control has been demonstrated during synthesis^36^, it is unclear if this is possible during dissolution.

## Discussion

We measured *in-situ* 3D displacement field dynamics in a single ZnO crystal during ultrafast laser excitation. The energy deposited by the laser excitation excites acoustic phonon modes, including both lattice breathing modes and shear modes. Using the 3D displacement field, we mapped the spatial distribution of the acoustic phonon modes and observed dynamics as a function of delay time, which were then compared to finite element simulations. To investigate the possibility of tuning particle shape and size during the experiment, we exposed a single ZnO crystal to dilute acid. The acid heterogeneously dissolves the crystal and changes both the shape and the size. Thus, acid etching could be used to investigate size and shape effects on excited phonon distributions at an XFEL. In general, our results pave the way for future studies of phonon mode propagation in crystals and, for example, how shape and size influence phonon scattering at the nanoscale.

## Methods

### ZnO synthesis

The synthesis of ZnO hexagonal-prism-shaped nanowires was carried out in a horizontal quartz tube furnace through solid-phase chemical vapor transport and deposition. A crucible containing the source material was placed in the center of the tube. This consists of a fine mixture of high-purity (99.9999%) 300 mesh graphite and zinc carbonate (ZnCO_3_·2Zn(OH)_2_·H2O) powder. Si substrates with (111) orientation were cleaned in acetone and propyl alcohol and placed in the downstream region. The system was subsequently purged with 500 s.c.c.m. (standard cubic centimeters per minute) of Ar carrier gas with an O_2_ content of 0.5–5% for 1 h. After this, the tube furnace was heated to 900 °C with the gas flow remaining. At 900 °C carbothermal reduction of ZnC released supersaturated Zn, which combines with O_2_ to form wurtzite ZnO in the cooler downstream region at 550 °C. The reaction proceeded for 30 min, after which the system was allowed to cool naturally. These crystals are the same that was used previously for BCDI studies^30, 37^.

### LCLS XPP Description

The experiments were performed at the x-ray pump probe instrument of the Linac Coherent Light Source using ~9.5 keV x-rays at a repetition rate of 120 Hz with approximately 80 fs full duration at half maximum (FDHM). Beryllium lenses were used to focus the x-rays to a 30 × 30 micron spot. The Beryllium lenses were positioned so that the sample position was out of the nominal focus, thereby reducing the intensity of the x-rays on the sample. The x-ray beam was attenuated to 5% of its maximum intensity to prevent crystal damage. A 2.3 megapixel CS-PAD detector with 110 µm square pixels was used to record the diffraction. A set of 60 2D diffraction patterns, each pattern consisting of the sum of 1000 pulses, was recorded at the ZnO (100) Bragg peak for each delay time. Filtering of the data was done ex post facto to remove saturated frames and blank shots. The remaining shots (~800) were then summed together to make the single frame. A Ti-sapphire laser with a wavelength of 266 nm, pulse length of 50 fs FDHM, and size of approximately 50 × 50 micron was used to induce acoustic phonons in the crystals. The incident fluence was varied from 1–10 mJ/cm^2^. The delay time was varied to probe the acoustic phonons stroboscopically.

### Phase retrieval procedure

The phase retrieval code is adapted from published work^38, 39^. The hybrid input-output^21, 40^ and error reduction algorithms were used for all reconstructions. A total of 1050 iterations, consisting of alternating 40 iterations of the hybrid input-output algorithm with 10 iterations of the error reduction algorithm, were run for 10 reconstructions beginning from random phases. The best reconstruction, quantified by the smallest sharpness metric, was then used in conjunction with another random phase start as a seed for another 10 random starts. The sharpness metric is the sum of the absolute value of the reconstruction raised to the 4^th^ power. 10 generations were used in this guided algorithm^41^. The LCLS computing resources were used for the reconstructions.

### Synchrotron BCDI experiment description

Experiments were performed at Sector 34-ID-C of the Advanced Photon Source at Argonne National Laboratory. A double crystal monochromator was used to select E = 8.919 keV x-rays with 1 eV bandwidth and longitudinal coherence length of 0.7 *μm*. A set of Kirkpatrick-Baez mirrors was used to focus the beam to 2 × 2 *μm* ^2^ (vertical × horizontal). The rocking curve around the ZnO (100) Bragg peak was collected by recording 2D coherent diffraction patterns with an x-ray sensitive area detector (Medipix2/Timepix, 256 × 256 pixels, each pixel 55 μm × 55 μm). It was placed a distance of 2 m away from the sample and an evacuated flight tube was inserted between the sample and the camera. A total of 61 patterns were collected for a single 3D rocking scan over a total angular range of $({\rm{\Delta }}\theta =\pm {0.2}^{\circ })$. Each 3D data set takes approximately 10 minutes.

### Finite element simulations of acoustic phonon modes

To compute acoustic phonon modes in the ZnO nanoparticle, we use a linear elasticity model to solve the following eigenvalue problem:

1 $$\nabla \cdot {\sigma }_{ij}=\lambda \rho {u}_{j}$$

where *λ* is the eigenvalue and *u*_*j*_, the elastic displacements, is the corresponding eigenvector (the Einstein summation convention is used here). The mass density, *ρ*, is taken as 5.605 g/cm^3^. The Cauchy stress tensor, *σ*_*ij*_, is determined following the constitutive relationship ${\sigma }_{ij}={C}_{ijkl}{{\epsilon }}_{kl}$, where *C*_*ijkl*_ is the fourth-order elastic stiffness tensor and the elastic strain, *ε*_*ij*_, is ${{\epsilon }}_{ij}=\frac{1}{2}(\frac{\partial {u}_{i}}{\partial {x}_{j}}+\frac{\partial {u}_{j}}{\partial {x}_{i}})$. We take *C*_1111_ = *C*_2222_ = 209.5 GPa, *C*_3333_ = 220.2 GPa, *C*_1122_ = 106.6 GPa, *C*_1133_ = *C*_2233_ = 96.69 GPa, *C*_1313_ = *C*_2323_ = 50.84 GPa, and *C*_1212_ = 51.46 GPa^42^. Phonon frequencies are calculated according to the formula $f=\sqrt{\lambda }/2\pi$. The nanoparticle is 640 nm long and 580 nm wide from the top to the bottom hexagonal faces. The eigenvalue problem is solved using FEniCS^43^ for the particle with free boundaries. The finite element mesh is generated with Gmsh^44^ with a characteristic element length of 12 nm, and optimized first with gmsh and then with Netgen^45^ algorithms.

### Acid etching procedure

35 mM nitric acid was prepared by diluting 37 wt.% nitric acid (McMaster-Carr) with deionized water. The acid was added dropwise to the sample and allowed to sit on the sample for the indicated time. At the end of this time, the sample was washed with water and allowed to dry. Drying times were shortened by using a small nitrogen flow over the surface of the sample.

### Data availability statement

The data reported in this paper are available upon request. All code, including the reconstruction algorithm, is also available upon request.

## Electronic supplementary material

Supplementary Movie
